# Nobiletin promotes fracture healing by modulating macrophage polarization: a review

**DOI:** 10.3389/fphar.2025.1724568

**Published:** 2026-01-06

**Authors:** Zhiqi Zhang, Shangyi Liu, Wenhao Li, Kangquan Shou

**Affiliations:** 1 Department of Burn, Three Gorges University Hospital of Traditional Chinese Medicine & Yichang Hospital of Traditional Chinese Medicine, Yichang, China; 2 Department of Orthopedics, the First College of Clinical Medical Sciences, China Three Gorges University and Yichang Central People’s Hospital, Yichang, China

**Keywords:** macrophage polarization, fracture, inflammatory, nobiletin, bone ununion

## Abstract

Fracture healing is a complex process in which various cells, including macrophages, are involved. M1 macrophages and the cytokines they secrete exacerbate local inflammation and inhibit fracture healing, whereas M2 macrophages inhibit inflammation and promote tissue repair. An imbalance in the ratio of M1 to M2 macrophages leads to delayed healing and nonunion of the fracture. Nobiletin has received increasing attention in promoting fracture healing. Thus, the aim of this review article was to investigate the effects of nobiletin on macrophages during fracture healing, with a major focus on the potential mechanisms and applications, and to provide a reference for further research on this compound.

## 1 Introduction

Delayed healing and nonunion of fractures are common, and the number of patients with these orthopaedical conditions continues to increase with the aging population annually. It has been reported that bone nonunion occurs in approximately 10%–15% of patients who undergo operations for fractures (Bowers and Anderson, 2024; Castillo et al., 2023). A systematic review of 111 studies from 15 countries revealed that the overall prevalence of tibial fracture nonunion currently stands at 6.8%. The prevalence varied by country, with 65 studies involving China finding that the prevalence of tibial nonunion was 4.7% (Tian et al., 2020). At present, surgery is considered the gold standard for treating delayed fracture healing and nonunion, and should be performed as soon as possible following a diagnosis of nonunion. During surgery, the original internal fixation is usually removed, the soft tissue embedded in the broken end of the fracture is cleaned, and the already closed medullary cavity is recanalized before bone grafting is performed. However, the surgical approach has the disadvantages of large trauma, numerous complications, and a high risk of infection. Therefore, exploring new treatment modalities for fracture nonunion has gradually become a research hotspot.

Monocytes/macrophages originate from progenitor cells. At the onset of inflammation, monocytes migrate into tissues and differentiate into macrophages in response to local cytokine stimulation. Macrophages, as part of the immune system, play a crucial role in the development and progression of inflammation, including the removal of foreign bodies, phagocytosis of apoptotic cells, presentation of antigens, and production of cytokines (Sica and Mantovani, 2012; Wynn et al., 2013). Activated macrophages are typically divided into two categories, namely, M1 and M2. M1 macrophages are mainly involved in pro-inflammatory responses, while M2 macrophages are involved primarily in anti-inflammatory responses (Yunna et al., 2020). In addition, M1 macrophages have potent anti-microbial and anti-tumor activity, mediate tumor-induced tissue damage, and impair tissue regeneration and wound healing (Kadomoto et al., 2021; Shapouri-Moghaddam et al., 2018). M2 macrophages have a strong phagocytic capacity, remove debris and apoptotic cells, promote tissue repair and wound healing, and have pro-angiogenic and pro-fibrotic properties (Wynn and Ramalingam, 2012; Braga et al., 2015). Thus, the balance of M1/M2 macrophage polarization is important for the maintenance of immune homeostasis. The presence of an excessively large number of M1 macrophages may lead to various diseases, including non-healing fractures (Gu et al., 2017). Promoting M1 to M2 polarization may be a new direction to promote fracture healing (Zhao S. J. et al., 2020; Pajarinen et al., 2019). While nobiletin (NOB) has been investigated in metabolic and neurodegenerative diseases, its application in bone repair—especially through immune regulation—is underexplored.

NOB is a polymethyl flavonoid extracted from *Citrus aurantium*. Previous studies have demonstrated that NOB exerts anti-inflammatory (Huang et al., 2023), anti-tumor (Huang et al., 2022), anti-neurodegenerative (Nakajima and Ohizumi, 2019), and antioxidant (Wang et al., 2022) effects. This compound has shown great potential for bone healing (Tominari et al., 2012; Rojasawasthien et al., 2023; Wang Y. et al., 2019; Pang et al., 2021). A few studies have also confirmed that NOB regulates macrophage polarization (Yang et al., 2022; Wang et al., 2021; Guo et al., 2012).

In this review, we delve into the phenomenon of macrophage polarization and its importance in the fracture healing process. Emphasis is placed on the disturbance of the M1/M2 macrophage balance, which can lead to an excessive inflammatory response triggered by pro-inflammatory cytokines and chemokines. In addition, we have performed an exhaustive analysis of the role of NOB in promoting fracture healing, particularly the mechanism through which it regulates macrophage polarization. An in-depth understanding of the mechanism of action of NOB will help develop new therapeutic strategies to address the problem of fracture nonunion.

## 2 Role of macrophages in fracture healing

### 2.1 Macrophage origin and polarization

In the past, it was thought that macrophages in tissues were derived from monocytes in the circulating blood. However, more recent research yielded evidence indicating that tissue macrophages have a dual origin, either as self-sustaining local macrophages or as monocyte-derived macrophages (Bajpai et al., 2018). Macrophages are found in most tissues of the body, where they maintain the normal functions of the organs; they are also involved in the metabolism of iron, bilirubin, calcium, lipids, and amino acids and help to regulate the levels of these substances in the body (Mosser et al., 2021).

M1 macrophages are induced by lipopolysaccharide (LPS) alone or in co-stimulation with T helper 1 cytokines and play a pro-inflammatory role, while M2 macrophages are induced by T helper 2 cytokines and have an anti-inflammatory function (Shapouri-Moghaddam et al., 2018). Depending on the inducing factor, M2 macrophages can be classified into four different subtypes, namely, M2a, M2b, M2c, and M2d. Their activators, secreted cytokines, and functions are shown in Table 1.

**TABLE 1:** Macrophage subpopulations and their functions.

Macrophage phenotype	Stimulus	Secreted cytokines	Function
M1	LPS, IFN-γ, IL-12, IL-23	TNF-α, IL-1α, IL-1β, IL-6, IL-12, 1L-23, CXCL9, CXCL10	Proinflammatory activity and tissue damage
M2a	IL-4, IL-13	TNF-a, IL-1α, IL-1β, IL-6, IL-12, IL-23, CXCL9, CXCL10, CXCL11, CXCL16, CCL5, TGF-β, IGF-1	Promotion of wound healing and fibrosis
M2b	TLR ligands, IL-1 β	IL-1β, TNF-α, IL-6, IL-10, CCL1	Anti-inflammatory and immunomodulatory properties
M2c	IL-10, TGF-β	IL-10, TGF-β, CCL16, CCL18, CXCL13	Phagocytosis, immunosuppression, angiogenesis and tissue fibrosis development
M2d	TLR antagonists	IL-10, VEGF	Angiogenesis and tumor progression

LPS, lipopolysaccharide; IFN-γ: interferon-γ; IL, interleukin; TNF-α, tumor necrosis factor-α; CXCL, chemokine (C-X-C motif) ligand; CCL, C-C motif chemokine ligand; TGF-β, transforming growth factor-β; IGF-1, insulin-like growth factor-1; TLR, toll-like receptor; VEGF: vascular endothelial growth factor.

Macrophages are highly plastic and can switch from one phenotype to another (Sica and Mantovani, 2012). There are limits to this plasticity. For example, Van den Bossche et al. (2016) found that mouse and human M1 macrophages failed to convert to M2 cells after interleukin 4 (IL-4) stimulation both *in vitro* and *in vivo*. Further studies identified the inhibition of M1-associated mitochondrial oxidative phosphorylation as a factor preventing the repolarization of M1 to M2 macrophages. An animal experiment showed that Atk kinase induces macrophage polarization to varying degrees, with Atk1 promoting M1 polarization and Atk2 promoting M2 polarization. In this process, the expression of miR-155 and its target CCAAT/enhancer-binding protein beta (C/EBPβ) appears to play a key role (Arranz et al., 2012). In addition, experimental evidence showed that M2 polarization of macrophages can be induced by downregulating JUN N-terminal kinase (JNK) phosphorylation in adipose tissue of obese rats (Oliveira et al., 2013). An *in vitro* experiment on human monocyte macrophages confirmed a regulatory effect of angiotensin-converting enzyme (ACE) on macrophage polarization. Moreover, subsequent mechanistic studies found that activation of AMPK phosphorylation increased AMPK-dependent ACE expression, promoting a decrease in the M1 phenotype or polarization towards the M2 phenotype (Kohlstedt et al., 2011). A large number of experiments confirmed that M1 to M2 polarization can be promoted through various pathways, such as the formyl peptide receptor 2/ALX-dependent (FPR2/ALX-dependent) AMPK/mechanistic target of rapamycin kinase (AMPK/mTOR) pathway (Xu et al., 2021), the transforming growth factor-β/SMAD (TGF-β/SMAD) pathway (Geng et al., 2023), the IL-4/signal transducer and activator of transcription 6 (IL-4/STAT6) signaling pathway (Yang et al., 2021), miR-934 (Zhao S. et al., 2020), and miR-223 (Dang and Leelahavanichkul, 2020).

Control of macrophage polarization is achieved by multilayered regulation of gene expression; macrophages stimulated by LPS differentially express approximately 4,500 genes compared with normal macrophages (Dalby et al., 2020). Epigenetic regulation (Murray, 2017; Foster et al., 2007; Ramirez-Carrozzi et al., 2006) and post-transcriptional regulation (Locati et al., 2020) together determine the macrophage polarization status; for example, microRNAs can modulate M1/M2 phenotypic switching by regulating target genes (Ambros, 2024; Zhong and Yi, 2016; Rückerl et al., 2012). Specific epigenetic features are associated with distinct macrophage differentiation states, and genome-wide analyses have shown that enhancers undergo rapid reorganization in LPS-induced macrophages to support pro-inflammatory phenotypes (Squadrito et al., 2012).

**FIGURE 1:**
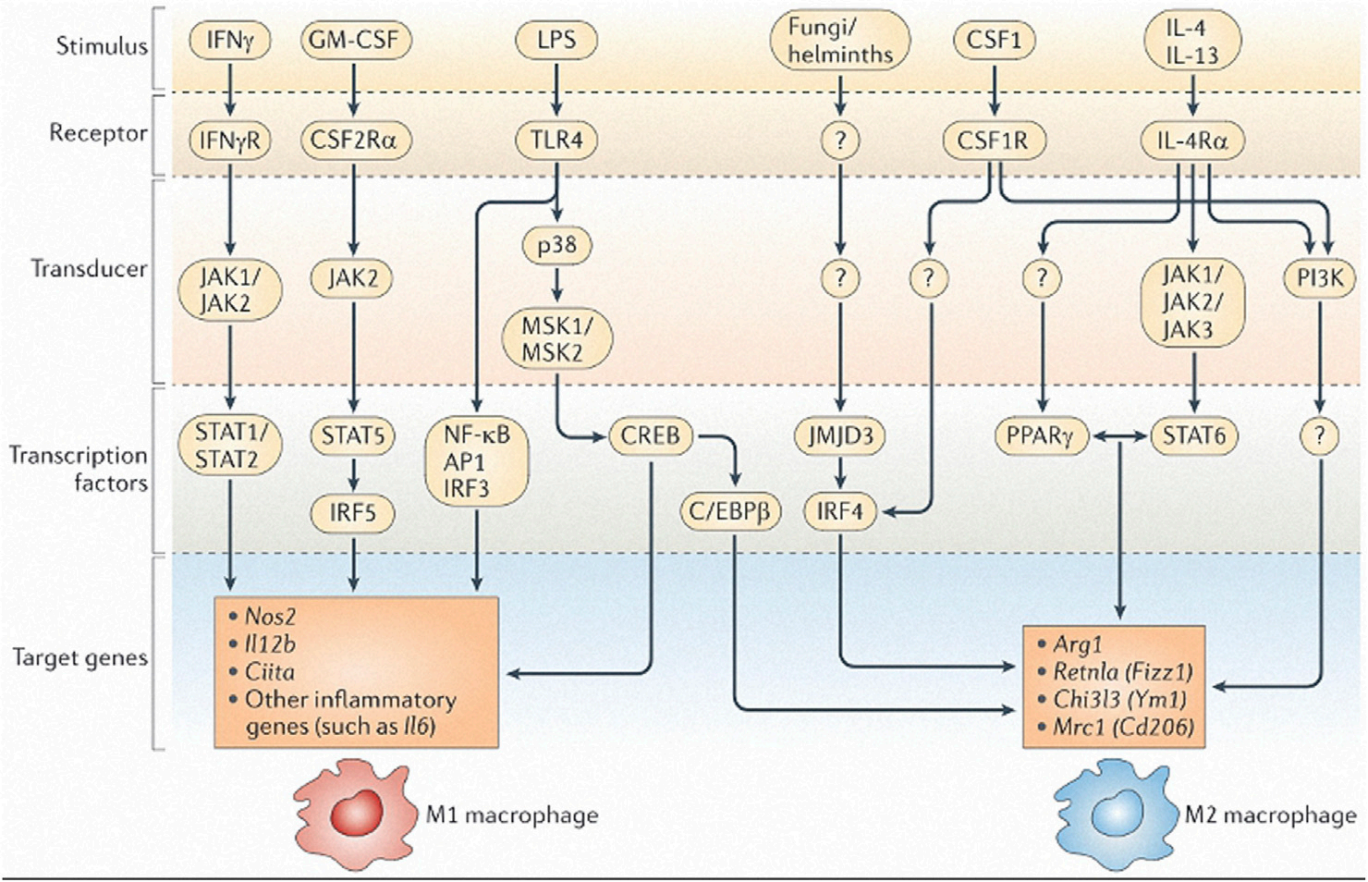
Signal transduction pathways to M1 and M2 macrophage polarization. Adapted from: Lawrence T, Natoli G. Transcriptional regulation of macrophage polarization: enabling diversity with identity. Nat Rev Immunol. 2011; 11(11):750–61.

Murray (Murray, 2017) summarized the molecular pathways involved in macrophage polarization; M2 polarization includes IL-4, IL-13, interleukin 4 receptor (IL-4R), STAT6, and key downstream transcription factors controlling M2 gene expression such as interferon regulatory factor 4 (IRF4), peroxisome proliferator activated receptor γ (PPARγ), PPARδ, and jumonji domain containing 3 (JMJD3). Loss of function of any of the genes coding for these factors results in either complete or substantial inhibition of the expression of M2 polarization genes, or marked reduction in the number of M2 macrophages. M1 polarization includes interferon-γ (IFN-γ), tumor necrosis factor (TNF), toll-like receptor (TLR), and IL-1R signaling as well as STAT1, IRF5, and nuclear factor-κB (NF-κB). In the specific transcription of macrophage polarization, the above molecules can induce gene promoters through different signaling pathways to determine the functional polarization of macrophages. Lawrence and Natoli (Lawrence and Natoli, 2011) summarized the signaling pathways stimulated by IFN-γ, granulocyte-macrophage colony stimulating factor (GM-CSF), LPS, colony stimulating factor 1 (CSF1), IL-4, and IL-13 that lead to M1 and M2 macrophage polarization (Figure 1).

### 2.2 Effect of macrophages on bone marrow mesenchymal stem cells (MSCs)

MSCs can differentiate into osteoblasts, adipocytes, chondrocytes, and vascular cells. Macrophages influence the migration, differentiation, proliferation, and apoptosis of bone marrow MSCs in addition to helping them differentiate into osteoblastic bone marrow MSCs, which in turn assists bone neogenesis. MSCs can also influence macrophage polarization and function. M1 macrophages contribute to the initial acute inflammatory phase and debris removal at the fracture site, whereas M2 macrophages secrete growth factors that support MSC-mediated bone formation during the later stages of fracture healing (Murray and Wynn, 2011).

Several past studies have shown that LPS-induced macrophages can promote the expression of large amounts of bone morphogenetic protein 2 (BMP2), runt-related transcription factor 2 (RUNX2), and alkaline phosphatase (ALP) by human MSCs *in vitro* (Omar et al., 2011; Guihard et al., 2012). Further mechanistic studies revealed that M1 macrophages produce tumor suppressor M (OSM), which promotes osteogenic differentiation by acting on the STAT3 signaling pathway in MSCs (Nicolaidou et al., 2012). However, the prevailing view at present is that cytokine production by M1 macrophages affects osteoblast differentiation (Toita et al., 2024; Gong et al., 2016) and the risk of osteoporosis (Dou et al., 2018; Vannella and Wynn, 2017). Large numbers of M1 macrophages inevitably lead to bone resorption and increased osteoclast activity (Souza and Lerner, 2013). In a study, the expression of RUNX2 and ALP was upregulated after culturing MSCs in conditioned medium using M2 macrophages. This observation may be related to the activation of STAT3 in MSCs by monocyte-produced OSM (Nicolaidou et al., 2012). An experiment in which macrophages were co-cultured with MSCs showed that a decrease in the M1/M2 ratio promoted MSC-mediated osteogenesis (Gong et al., 2016). Moreover, consistent results were obtained in human cells (Fernandes et al., 2013); IL-4-treated human umbilical cord blood macrophages were able to stimulate osteogenic differentiation of MSCs.

Taken together, these studies suggest that both M1 and M2 macrophages affect the osteogenic differentiation of MSCs through different ways. Specifically, M1 macrophages promoted early and mid-stage osteogenesis, but did not increase bone mineralization. In contrast, M2 macrophages promoted late osteogenesis and promoted bone mineralization.

### 2.3 Effect of macrophages on osteoblasts

Osteoblasts are derived from MSCs and play important roles in the formation, synthesis, secretion, and mineralization of bone (Harada and Rodan, 2003). Macrophages are essential for the maintenance of osteoblast function. Following the timely transition of macrophages from the M1 phenotype to the M2 phenotype, osteoblasts exhibited enhanced proliferation, adhesion, and extracellular mineralization, as well as enhanced expression of osteogenesis-related genes (RUNX2, ALP, collagen type I alpha 1 chain [COL1A1], opsin [OPN], and osteocalcin [OCN]) (You et al., 2022).

The regulation of osteoblasts by macrophages is largely dependent on specific cytokines secreted by macrophages. When osteoblasts (MC3T3) were co-cultured with macrophages, inactivated M0 as well as M1 and M2 macrophages promoted osteogenesis. Nevertheless, treatment of M1 with IL-4 (which polarized M1 to M2 macrophages) increased the osteogenic differentiation capacity of MC3T3 cells, and treatment of MC3T3 cells with direct administration of IL-4 did not show osteogenic capacity. These findings suggested that the increase in osteogenic capacity was due to the presence of M2 macrophages and the secretion of potential osteoclastogenic cytokines (Loi et al., 2016). M2 macrophages are indispensable for osteoblast differentiation and osteogenesis. The mRNA expression of BMP2 and osterix (OSX) increases after M2 macrophage polarization, thus promoting osteogenesis. In addition, M2 macrophages secrete BMP2, which activates the phosphorylation of SMAD1 and induces RUNX2 translocation to the nucleus by binding to BMP receptors, thereby leading to increased expression of ALP and OCN in the cell. This induces an increase in ALP and OCN expression in the cells and promotes osteogenesis (Wu et al., 2022; Cho et al., 2022).

### 2.4 Effect of macrophages on osteoclasts

Osteoclasts are large multinucleated cells that differentiate from a monocyte/macrophage cell lineage via macrophage colony-stimulating factor (M-CSF) and NF-κB ligand receptor-activating factor (RANKL); anti-tartrate acid phosphatase and histone K are major hallmarks of these cells (Ono and Nakashima, 2018). Osteoclasts play an important role in all stages of osteogenesis (Tosun et al., 2022). Nonetheless, the role of osteoclasts in the scab-shaping stage is controversial; some studies suggest that osteoclasts play no role in this stage, whereas others imply that they are essential at this stage and that osteoblasts and osteoclasts work together to facilitate the process of resorption of the scab and bone formation (Zhang et al., 2023). As central regulators of bone homeostasis, macrophages sense stimuli from osteoblasts and osteoclasts and subsequently respond with the appropriate polarization state (Horwood, 2016).

There is increasing evidence that secretions from M1 macrophages promote osteoclast differentiation and maturation, whereas M2 macrophages produce the opposite effect. This difference may be related to the secretion of cytokines and chemokines by macrophages of different polarization states (Sun et al., 2021). Hu et al. (2023) reviewed the signaling pathways affected by these cytokines. They reported that TNF-α secreted by M1 macrophages activates the NF-κB and phosphoinositide-3-kinas/AKT (PI3K/AKT) signaling pathways. Additionally, IL-1β increases the expression of iOS, insulin like growth factor 2 (IGF2), stromal cell-derived factor 1 (SDF1), and C-X3-C motif chemokine ligand 1 (CX3CL1). Furthermore, IL-6 promotes osteoclast differentiation and activity through the Janus kinase/STAT (JAK/STAT) signaling pathway. IL-4 secreted by M2 macrophages inhibited osteoclast differentiation by downregulating RANKL expression in osteoblasts and inhibiting NF-κB.

### 2.5 Role of macrophages in vascular endothelial cells

In the skeletal system, blood vessels act as a conduit system for transporting oxygen, nutrients, metabolic wastes, or cells, and provide multifunctional signaling molecules that regulate bone development, regeneration, and remodeling. Formation of new blood vessels at the fracture site restores hypoxia and nutrient deprivation in the early stages of the fracture, whereas in the later stages of the fracture, MSCs circulating in the peripheral blood are mobilized during fracture healing and may contribute to fracture repair (Iwakura et al., 2013). In addition to their direct action on osteocytes, macrophages promote bone and soft tissue repair by facilitating the regeneration of blood vessels around fractures (Zhang et al., 2020).

**FIGURE 2:**
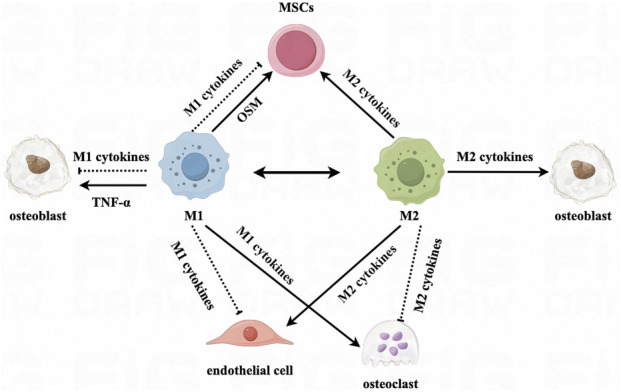
Effects of polarized macrophages on mesenchymal stem cells, osteoblasts, osteoclasts, and endothelial cells.

In an experiment involving co-culture of M1 macrophages and vascular endothelial cells, it was shown that exosomes containing miR-155 produced by M1 macrophages exacerbated endothelial–mesenchymal transition in endothelial cells via the NF-κB pathway. Moreover, mitochondrial dysfunction was observed, leading to an increase in reactive oxygen species (ROS), which could damage vascular endothelial cells (Ge et al., 2021). Similarly, a shift in macrophage phenotype towards M1 after treatment of M2 macrophages with *Treponema pallidum* inhibited angiogenesis in human umbilical vein endothelial cells (Wang Y. J. et al., 2023). In contrast, M2 macrophages showed a protective effect on the vascular endothelium, and the addition of *Astragalus* after high glucose-induced macrophage M1 polarization revealed a shift in macrophage phenotype towards M2. This resulted in a reduction of ROS production and a protective effect on the vascular endothelium; however, the mechanism underlying this process was not elucidated (Sha et al., 2023). The prolyl hydroxylase 2 (PHD2) gene is an oxygen sensor that rescues the blood supply by regulating the formation and shape of blood vessels in the presence of insufficient oxygen. In PHD2-deficient mice, M2 macrophages regulate astrogenesis and homeostasis through the secretion of angiogenic factors that promote the recruitment and value-addition of smooth muscle cells (Takeda et al., 2011). It has also been demonstrated that the ability of M2 macrophages to promote angiogenesis is associated with the fibroblast growth factor 2 (FGF2) signaling pathway and the platelet growth factor (PlGF) signaling pathway (Jetten et al., 2014) (Figure 2).

## 3 Role of NOB in fracture healing

### 3.1 Biological activity of NOB

NOB is usually found in the stems, leaves, and pericarp of plants in the Rosaceae and Euphorbiaceae families, and has significant biological activities (e.g., antioxidant, anti-inflammatory, anticarcinogenic, immune enhancement, and reduction of cardiovascular disease risk) (Jun, 2019; Jingxiang et al., 2011). A large number of *in vivo* and *ex vivo* experiments in animals have confirmed that NOB can exert anti-inflammatory effects and immune-modulation through inhibition of the IL-23/IL-17 axis, as well as modulate endoplasmic reticulum stress, the C-C motif chemokine ligand 2/C-C motif chemokine receptor 2 (CCL2/CCR2) signaling axis, and the forkhead box O3A/sirtuin 1 (FOXO3A/SIRT1) pathway (Shiping et al., 2024; Qing L. et al., 2024; Jinxian et al., 2023; Qing Y. et al., 2024).

Although NOB has shown good pharmacological activity in *ex vivo* and *in vivo* experiments in animals, NOB-based drugs have not been developed due to its poor water solubility, poor permeability, low bioavailability, and increased first-pass metabolism (Kesharwani et al., 2020). The low bioavailability is currently the greatest challenge in research on NOB. Its poor water solubility results in very low bioavailability (<1%) after oral administration, which is currently the main route (Onoue et al., 2013). Therefore, local administration may be an option in bone regeneration, and the development of a carrier with some strength and the ability to load NOB to improve its bioavailability may be the way forward.

To further address the issues of poor absorption and low local bioavailability of NOB during fracture healing, targeted local delivery systems are crucial, and tissue engineering technologies hold significant potential (Mittwede et al., 2018). Biodegradable polymer scaffolds are preferred (Dai et al., 2016). These scaffolds possess mechanical strength matching early fracture sites, can efficiently encapsulate NOB, and degrade gradually to release NOB in a sustained manner—avoiding rapid drug clearance and maintaining stable local drug concentrations. Bioactive ceramic scaffolds are also suitable (Li et al., 2025), as they act as NOB carriers and mimic natural bone composition to promote osteoconduction, synergizing with NOB to enhance healing. In addition to scaffolds, hydrogels are also an excellent option. Hydrogels offer high flexibility and moldability, allowing them to easily fill irregular areas of bone defects. They tightly adhere to bone tissue to bridge gaps, thus ensuring targeted NOB release (Wu et al., 2020).

### 3.2 NOB for bone regeneration

The occurrence of a fracture occurs is followed by the hematoma inflammation mechanism period. The hematoma of the fracture can condense into a blood clot in 6–8 h after the injury, and numerous inflammatory cells infiltrate to solidify the hematoma. Necrotic tissue and osteoblasts in fracture breaks secrete growth factors to promote the aggregation and differentiation of MSCs to osteoblasts. At the same time, osteoclasts begin to develop and secrete collagen fibers, which facilitate the joining of the fracture breaks and are recruited to resorb the fractured fragments. This process is completed within approximately 2 weeks after the fracture. Subsequently, during the primary bone scab-formation phase, osteoblasts synthesize and secrete a large amount of bone matrix, causing a large amount of bone-like tissue to encircle the fracture end to form internal and external bone scabs. This phase typically lasts for 3–6 months. During the remodeling phase, osteoblasts at the fracture end promote the transformation of the scab to the lamellar bone, and osteoclasts remove necrotic bone, thus remodeling the hard scab. Consequently, the fracture breaks form a strong bony connection and the bone is restored to resemble its state prior to the injury (Bahney et al., 2019). The ability of NOB in osteogenesis has been demonstrated; below, we provide an overview of the mechanisms underlying the effects of NOB on osteogenesis.

#### 3.2.1 NOB directly promotes bone regeneration

Numerous tissues or cells, including MSCs, osteoblasts, osteoclasts, etc., are directly involved in this process fracture healing. In this section, we summarize recent evidence regarding the effects of NOB on these cells. The NF-κB family of transcription factors plays an important role in bone development and skeletal homeostasis. It has been demonstrated that NOB reduces the induction of classical NF-κB target genes by TNF-α, thereby enhancing osteoblast activity (Rojasawasthien et al., 2023). An experiment showed that oral administration of a NOB-containing combination restored femoral bone loss induced by estrogen deficiency in ovariectomized mice; however, the specific mechanism underlying this effect remains unclear (Matsumoto et al., 2018). Previous experiments demonstrated that NOB inhibits bone resorption by inhibiting NF-κB-dependent prostaglandin E synthesis in osteoblasts (Harada et al., 2011). NOB metabolites can also inhibit bone-resorbing osteoclast differentiation and function via the NF-κB pathway (Hirata et al., 2023). In addition to the NF-κB pathway, NOB inhibited the differentiation of bone marrow-derived macrophages to osteoclasts via the RANKL-induced mitogen-activated protein kinase (MAPK) signaling pathway, thereby ameliorating bone loss (Wang Y. et al., 2019). In an *in vitro* experiment with human cells, it was confirmed that NOB promotes osteogenic differentiation of human osteoblasts through activation of the BMP2/RUNX2 signaling pathway (Pang et al., 2021). Thus far, only a few experiments involving human cells have been conducted. Compared with humans, animals are smaller and have a relatively simpler bone structure and lower bone strength; moreover, the biochemical evaluations as well as the toxicity evaluations and pharmacokinetics of human and animal cells differ considerably. These differences result in a significant difference in the Chuan. The limitations of animal experimental studies of *Chenopodium album* are large, and this study may be important for follow-up investigations.

#### 3.2.2 Regulatory effects of NOB on macrophage polarization

Both innate and adaptive immune processes are essential at all stages of fracture healing, with specific cell-mediated immune functions clearing necrotic tissue, promoting angiogenesis, and initiating repair during the initial inflammatory phase after injury (Einhorn and Gerstenfeld, 2015). The expression of cytokines with inflammatory and immune functions varies during different stages of fracture healing and plays different roles. During acute inflammation after injury, TNF-α and IL-6 recruit cells required for tissue regeneration, and their complete absence delays the differentiation of bone-derived MSCs (Gerstenfeld et al., 2003; Glass et al., 2011). As immune cells, macrophages play an important role in fracture healing (Wang Y. et al., 2023), and promoting macrophage M2 polarization and decreasing M1 polarization exerts a significant effect on fracture healing.

In a study of ulcerative colitis, NOB binding to the estrogen receptor 1 (ESR1) inhibited the LPS-induced expression of inflammatory factors in RAW264.7 cells (Liu et al., 2021). In a study of nonalcoholic liver disease, NOB inYanghibited NLR family pyrin domain containing 3 (NLRP3) inflammatory vesicle assembly and reduced inflammatory factors produced by M1 macrophages *in vitro* and *in vivo*; nevertheless, the exact mechanism is unknown (Yang et al., 2022). Another study showed that NOB inhibition of nonalcoholic liver disease was mediated by retinoic acid-related orphan receptor α (RORα) modulation of Kruppel like factor 4 (KLF4) expression in macrophages, which reduced the number of M1 macrophages and expression of pro-inflammatory cytokines (Wang et al., 2021). A combination of NOB and docosahexaenoic acid synergistically inhibited the LPS-induced production of nitric oxide in RAW264.7 cells without cytotoxicity. This process may be related to the inhibition of extracellular signal-related kinase (ERK) and p38 phosphorylation and NF-κB nuclear translocation (Nishi et al., 2024). An experiment demonstrated that NOB attenuates LPS-induced inflammation by activating IL-6/STAT3/FOXO3A-mediated autophagy (Rong et al., 2021). Another experiment showed that NOB-induced inhibition of AP-1, NF-κB, and cAMP-response element-binding protein (CREB) activation suppressed the LPS-induced expression of cyclooxygenase-2 (COX-2) in RAW264.7 cells (Murakami et al., 2005). In addition, metabolites of NOB inhibited the expression of inflammatory factors in LPS-induced RAW264.7 cells (Li et al., 2007; Wu et al., 2015a; Wu et al., 2015b).

Furthermore, NOB induced macrophage polarization from an M1 to an M2 phenotype, and a study using a methionine and choline deficiency-induced model of nonalcoholic steatohepatitis demonstrated that NOB markedly attenuated hepatic injury and fibrosis in mice. This effect may be attributed to an elevation in the expression of KLF4 in macrophages to facilitate M2 macrophage polarization (Wang et al., 2021). In addition, in *ex vivo* experiments in mice with ulcerative colitis, NOB regulated NLRP3 inflammasome assembly, thereby promoting M2 macrophage polarization (Yang et al., 2024).

#### 3.2.3 Effects of NOB on blood vessels

**FIGURE 3:**
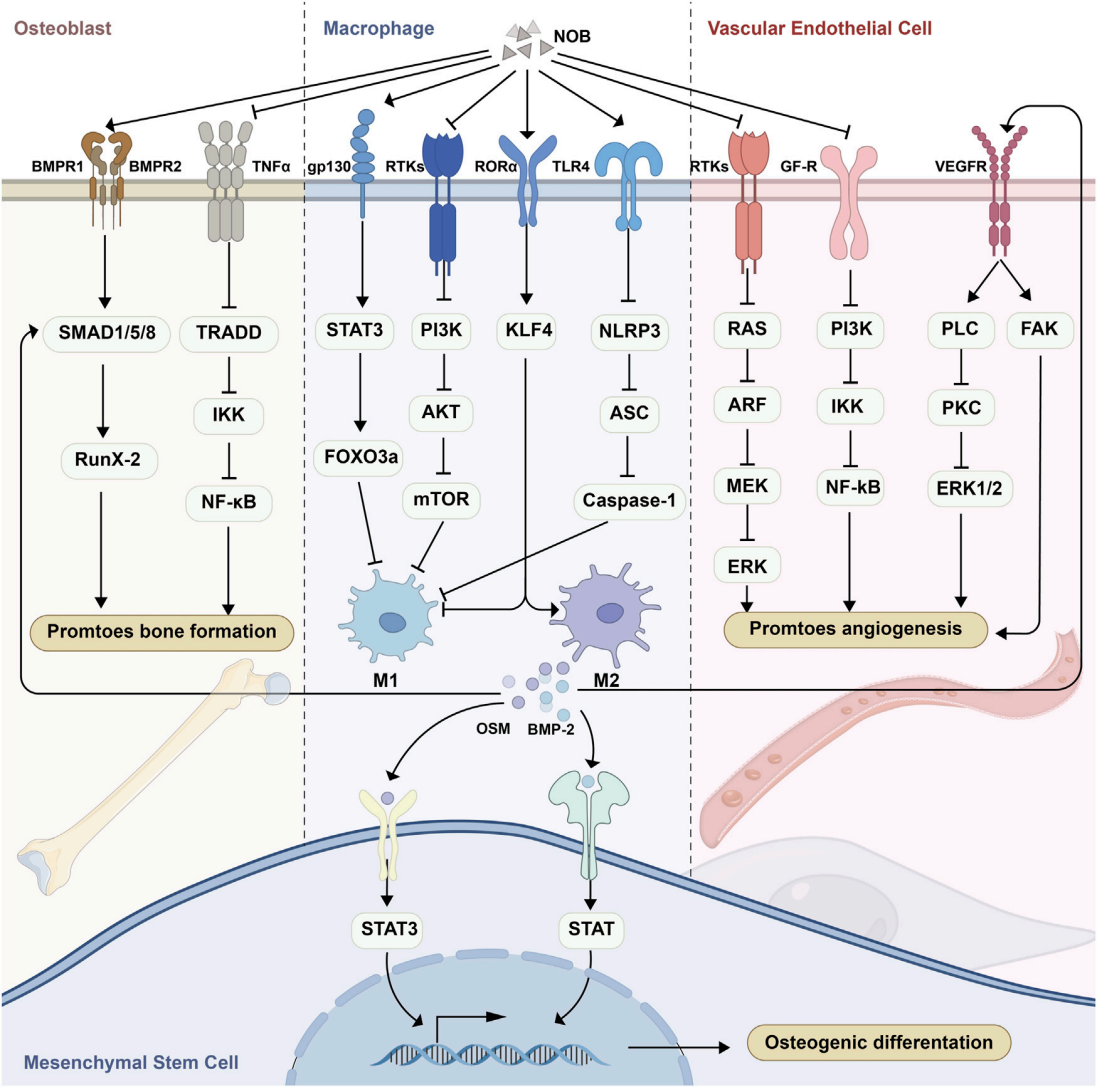
Schematic representation of the effects of NOB in promoting fracture healing. MSCs, mesenchymal stem cells; NOB, nobiletin.

Bone tissue has a rich blood supply. Blood vessels deliver nutrients, growth factors, osteoblasts, osteoclasts, etc. to the damaged area and carry away metabolic waste, thus providing a stable internal environment for fracture healing. A study showed that NOB attenuates vascular endothelial damage from iron overload via the ROS/asymmetric dimethylarginine/dimethylarginine dimethylaminohydrolase 2/endothelial nitric oxide synthase/nitric oxide (ROS/ADMA/DDAHII/eNOS/NO) pathway (Wang et al., 2020). In addition, NOB can inhibit vascular inflammation via the NF-κB/MAPK pathway (Yang et al., 2019) (Figure 3).

## 4 Conclusion and perspectives

Delayed healing and non-healing of fractures are major challenges for orthopedic surgeons. Currently, surgery and physical therapy are the main treatment methods. However, these approaches are limited by long treatment periods, poor quality of life, and postoperative complications that cannot be ignored. With the development of medical technology, an increasing number of biological agents are applied in clinical practice (Conway et al., 2014). NOB is a polymethyl flavonoid that promotes fracture healing, inhibits bone resorption, and promotes neovascularization at the fracture site through various pathways *in vivo* and *in vitro*.

However, current evidence supporting the role of NOB in fracture healing is limited to *in vitro* cell experiments and *in vivo* animal studies. This constraint poses substantial risks to direct clinical translation, rather than merely technical limitations of the models themselves. Firstly, there is a high risk of efficacy non-reproducibility in humans. Animal models differ from humans in the regulatory mechanisms of bone healing; specifically, humans have more complex systemic hormone regulation, richer vascular innervation in fracture sites, and slower bone turnover cycles compared with animals (e.g., humans require 3–6 months for fracture union, while rodents only require 2–4 weeks). These differences indicate that the ability of NOB to promote osteoblast proliferation or inhibit osteoclast activity observed in animals may not be replicated in humans. For example, the effect of NOB on accelerating callus formation in mice may not be observed in human patients with age-related osteoporosis or comorbidities such as diabetes.

Secondly, there is a critical risk of dose conversion bias when extrapolating research findings from animals to humans. Preclinical studies determine “effective doses” based on animal body weight or organ size. nevertheless, humans and animals metabolize NOB through distinct pathways; humans have higher activity of liver enzymes involved in drug clearance, leading to lower bioavailability and shorter half-life of NOB compared with animals. Using the animal-derived dose directly in humans may result in two adverse outcomes: the dose could be excessively low to achieve therapeutic effects on fracture healing, or excessively high, thereby increasing the burden on liver and kidney metabolism. Therefore, it is not possible to determine a safe and effective dose for humans based solely on preclinical data.

Thirdly, the use of preclinical data cannot avoid the risk of safety evaluation failure in clinical settings. Animal toxicology studies typically focus on acute toxicity and major organ damage however, fracture healing in humans requires long-term drug intervention (at least 1–3 months). Animals may not exhibit chronic toxicities that could emerge in humans, such as cumulative bone marrow suppression, gastrointestinal mucosal damage, or drug-drug interactions. Thus, the “safety” confirmed in animals does not guarantee the absence of unexpected adverse events in clinical practice.

These clinical translation risks—rather than mere model limitations—highlight the inherent gap between preclinical findings and human application. Therefore, future research must prioritize well-designed clinical trials to verify the efficacy of NOB in promoting human fracture healing. More importantly, the conversion risks should be addressed by optimizing the dose, confirming long-term safety, and exploring suitable administration routes for different patient populations. Overall, macrophage polarization (particularly M2 polarization) enhances fracture healing through an interaction between macrophages and MSCs (Pajarinen et al., 2019; Cho et al., 2014), which promotes osteoblast differentiation and activity and inhibits osteoclast activity. NOB can promote M2 polarization of macrophages *in vitro* and *in vivo* through a variety of pathways (Yang et al., 2022; Wang et al., 2021; Guo et al., 2012). We think NOB exerts its effects through this mechanism; nevertheless, this hypothesis must be tested in real broken bones. None of the studies discussed above were conducted in a fracture setting; thus, further investigation in a fracture setting is warranted to verify the currently available evidence.

The specific mechanism by which NOB promotes fracture healing is currently unclear, with the main studies currently focusing on the effects on osteoblasts. Macrophages are involved in the whole process of fracture healing and are important for bone regeneration. Nonetheless, no study to date has focused on the effect of NOB on macrophage polarization (especially M2 polarization) in fractures. Our subsequent studies will delve deeper into this aspect to elucidate the mechanism underlying the action of NOB. In addition, we need to overcome the problems of poor water solubility, low bioavailability, and first-pass metabolism of NOB. We think it is possible to design a scaffold that can increase the water solubility of NOB, achieve its slow release over a long period of time at the fracture site, and improve its biocompatibility. The development of such a scaffold may be able to solve the abovementioned problems, and provides a direction for the subsequent study of NOB.

## Abbreviations

ACE, Angiotensin-converting enzyme; ALP, Alkaline phosphatase; CREB, CAMP-response element-binding; ERK, Extracellular signal-related kinase; JNK, JUN N-terminal kinase; MAPK, Mitogen-activated protein kinase; MSC, Mesenchymal stem cells; PlGF, Platelet growth factor; ROS, Reactive oxygen species; TLR, Toll-like receptor; TNF, Tumor necrosis factor.
